# Comparative genomics of *Bacillus cereus sensu lato* spp. biocontrol strains in correlation to *in-vitro* phenotypes and plant pathogen antagonistic capacity

**DOI:** 10.3389/fmicb.2023.996287

**Published:** 2023-02-09

**Authors:** Maya Moshe, Chhedi Lal Gupta, Rakeshkumar Manojkumar Jain, Noa Sela, Dror Minz, Ehud Banin, Omer Frenkel, Eddie Cytryn

**Affiliations:** ^1^Institute of Soil, Water and Environmental Sciences, Agricultural Research Organization, Rishon-Lezion, Israel; ^2^Institute of Plant Pathology and Weed Research, Agricultural Research Organization, Rishon-Lezion, Israel; ^3^The Mina and Everard Goodman Faculty of Life Sciences, Bar-Ilan University, Ramat Gan, Israel

**Keywords:** biocontrol agent, chitinase, comparative genomics, phytopathogen, secondary metabolites, zwittermicin

## Abstract

*Bacillus cereus sensu lato* (Bcsl) strains are widely explored due to their capacity to antagonize a broad range of plant pathogens. These include *B. cereus* sp. UW85, whose antagonistic capacity is attributed to the secondary metabolite Zwittermicin A (ZwA). We recently isolated four soil and root-associated Bcsl strains (MO2, S−10, S-25, LSTW-24) that displayed different growth profiles and *in-vitro* antagonistic effects against three soilborne plant pathogens models: *Pythium aphanidermatum* (oomycete) *Rhizoctonia solani* (basidiomycete), and *Fusarium oxysporum* (ascomycete). To identify genetic mechanisms potentially responsible for the differences in growth and antagonistic phenotypes of these Bcsl strains, we sequenced and compared their genomes, and that of strain UW85 using a hybrid sequencing pipeline. Despite similarities, specific Bcsl strains had unique secondary metabolite and chitinase-encoding genes that could potentially explain observed differences in *in-vitro* chitinolytic potential and anti-fungal activity. Strains UW85, S-10 and S-25 contained a (~500 Kbp) mega-plasmid that harbored the ZwA biosynthetic gene cluster. The UW85 mega-plasmid contained more ABC transporters than the other two strains, whereas the S-25 mega-plasmid carried a unique cluster containing cellulose and chitin degrading genes. Collectively, comparative genomics revealed several mechanisms that can potentially explain differences in *in-vitro* antagonism of Bcsl strains toward fungal plant pathogens.

## 1. Introduction

The ban on many chemical pesticides has facilitated interest in discovery and application of bacteria (termed biocontrol agents) that antagonize soilborne plant pathogens. These bacteria protect plants from pathogens through a variety mechanisms that include niche exclusion (Wang et al., 2021), metabolic competition (Spadaro et al., 2010), production of siderophores (Yu et al., 2010; Li et al., 2014), secretion of chitinases that target the chitin components of fungal cell walls (Veliz et al., 2017), antibacterial and antifungal compounds (Raaijmakers et al., 2002; Ongena and Jacques, 2008), and induction of plant resistance (Pieterse et al., 2014). Secondary metabolites (SM), which include siderophores and antibiotics play a pivotal role in the antagonistic capacities of biocontrol agents, but the scope and the specific role of these compounds in different strains are not well understood (Braga et al., 2016).

*Bacillus cereus sensu lato* (Bcsl) is a phylogenetically related group that includes the well-studied member’s *B. cereus sensu stricto* (*s.s.*), *B. anthracis*, and *B. thuringiensis* (Stenfors Arnesen et al., 2008; Ehling-Schulz et al., 2019; Bianco et al., 2021; Carroll et al., 2021). Several of these strains produce a variety of biologically active plant-pathogen antagonizing molecules, and have thus been explored as potential biocontrol agents (Silo-Suh et al., 1994; Kumar et al., 2014a). *Bacillus cereus s.l.* strain UW85 (ATCC 53522) has been extensively explored as a biocontrol agent due to its *in-vitro* and *in-planta* capacity to antagonize various fungal and oomycetes pathogens, which is at least partially facilitated by the antimicrobials ZwA and kanosamine (Silo-Suh et al., 1994).

Bcsl strains frequently harbor multiple plasmids, including mega-plasmids larger than 100 kb (Zheng et al., 2013). These mega-plasmids have been primarily explored in obligatory and opportunistic *B. anthracis* and other *B. cereus s.l.* strains that carry genes and operons encoding for virulence factors (i.e., *cya*, *lef*, *pagA*, haemolysin BL, *capABCDE* and *cesABCD*; Okinaka et al., 1999; Hoton et al., 2005). In addition, certain *B. thuringiensis* strains harbor mega-plasmids with genes encoding insecticidal (Cry and Cyt) toxins that are used commercially to control different insect pests (Gillis et al., 2018). Recently, whole-genome sequencing revealed that the biosynthetic gene cluster encoding ZwA and Kanosamine in certain Bcsl strains is also situated on a mega-plasmid (Kevany et al., 2009; Lozano et al., 2016; Lechuga et al., 2020), suggesting that Bcsl strain mega-plasmids play a role in ecological adaptation and antagonism of plant pathogens.

The objective of this study was to identify Bcsl strain mechanisms potentially involved in antagonizing soilborne phytopathogens and pinpoint specific mechanisms that are unique to individual strains that may explain differences in their *in-vitro* antagonistic capacity. This was achieved by combining long-and short-read (i.e., Oxford Nanopore Minion and Illumina) whole genome sequencing, which enables assembly of complete chromosomes and plasmids (Arredondo-Alonso et al., 2017; George et al., 2017). Concomitant to whole genome sequencing, the five Bcsl strains (four isolated in our lab from different soils around Israel, and *B. cereus* strain UW85) were screened against the soilborne pathogens *Pythium aphanidermatum* (oomycetes), *Rhizoctonia solani* (basidiomycetes) and *Fusarium oxysporum* (ascomycetes) using both whole cell and cell extract antagonistic assays.

## 2. Materials and methods

### 2.1. Bacterial and fungal strains and growth conditions

*Bacillus cereus s.l.* UW85 (coined UW85), originally isolated from alfalfa roots in Wisconsin, USA, was purchased from the American Type Culture Collection (serial number ATCC 53522). *Bacillus cereus s.l.* S-25 (coined S-25) and *B. cereus s.l.* S-10 (coined S-10) were isolated from a clay-rich wheat field soil, and *B. cereus s.l.* MO2 (coined MO2) was isolated from the roots of a *Moringa oleifera* tree within the Volcani Institute campus of the Agricultural Research Organization (ARO) in Rishon Lezion, Israel. *Bacillus cereus s.l.* LSTW-24 (coined LSTW-24) was isolated from a sandy soil from the coastal plain of Israel. The studied Bcsl strains were grown on Luria-Bertani (LB) broth or agar and incubated at 30°C overnight, with or without shaking at 180 rpm. Their antagonistic effects was tested against three model soilborne pathogens: *R. solani* anastomosis group AG4, isolated from a tomato plant in the Western Negev, Israel, whereas *P. aphanidermatum* strain P88 and *F. oxysporum* f.sp. *radicis cucumerinum* strain Hazera were isolated from cucumber plants in the Hefer Valley, Israel. The three model pathogens were routinely grown on Potato Dextrose Agar (PDA, DIFCO, France), which was amended with 250 mg/l chloramphenicol (PDA+, Sigma, Israel) for growth of *R. solani* and *F. oxysporum*.

### 2.2. Evaluation of *in-vitro* antifungal activity

The *in-vitro* antagonistic activity of the five Bcsl group strains against the three model soilborne phytopathogens was evaluated using a standard dual culture assay. Briefly, we streaked an overnight bacterial suspension in the middle of a 9 cm Petri dish containing 50% LB agar, and incubated it at 30°C for 3 days in a temperature controlled incubator (Tuttnauer, Israel). Subsequently, a 5 mm diameter mycelial disk from the actively growing margin of one of the three pathogens described above was placed on opposite sides of the Petri dish and further incubated at 25°C. The inhibition zone between the bacteria and the pathogen, and the area of the pathogen mycelium were measured daily for 3 to 14 days after initial inoculation, depending on the rate of pathogen growth. Two criteria were considered when evaluating the *in-vitro* antagonistic capacity:

(1) Mycelia area - calculated as the proximal area of an ellipse according to the following formula:


$$\pi *\left(\frac{D1}{2}*\frac{D2}{2}\right)$$

where *D*1 is the long diameter and *D*2 is the short diameter.

(2) Inhibition zone - calculated as the minimal distance between the pathogen and the bacteria. A schematic diagram of both criteria is shown in Supplementary Figure S1.

### 2.3. Evaluation of *in-vitro* cell free supernatant antifungal activity

The five Bcsl strains were inoculated in 100 mL of 50% LB medium in 250 mL Erlenmeyer flasks and incubated at 30°C with shaking at 130 rpm for 6 days in a Witeg model WIS-30R shaking incubator (Witeg Labortechnik GMBH, Germany). Starting from the third day, 1 mL of culture from each flask was centrifuged and filtered through 0.22 µm, 22mm membranes and the cell-free supernatants (CFS) were stored at 4°C. PDA plates were inoculated with 4 mm diameter agar plugs containing one of the three model pathogens, and 50 μL of CFS (or 50% LB medium used as a control) was pipeted into aseptically created holes in the agar placed at a distance of 0.5 cm from *F. oxysporum* plug*,* 1 cm from *R. solani* plug and 1.5 cm from *P. aphanidermatum* plug. The plates were incubated at 25°C and inhibition was inspected after 1–3 days, depending on the pathogen growth rate. The experiment was conducted three times.

### 2.4. Evaluation of *in-vitro* chitinase and cellulase activities and genome screening for associated genes

Approximately 15 μL of an overnight culture of the five Bcsl strains was pipetted onto agar plates containing M9 minimal medium mixed with 0.4% colloidal chitin as a sole carbon source (Kuddus and Ahmad, 2013), and subsequently incubated for 20 days at 30°C. Chitinase activity was estimated by measuring the clearing zone around the bacterial colonies, calculated by subtracting the halo area from the area of the bacterial colony.

In tandem, we mined the five Bcsl genomes for genes associated with chitin metabolism (chitinases and chitin binding proteins) using RAST and BLASTX together with the web server dbCAN2, for carbohydrate-active enzyme (CAZyme) annotation (Zhang et al., 2018; Drula et al., 2022). The chitinase-associated genes sequences of the five Bcsl strains are shown in Supplementary Table S3.

The cellulose-degrading capacity of the five Bcsl strains was estimated by plating the strains on cellulose-amended Congo-Red agar media composed of: KH_2_PO_4_ 0.5 g, MgSO_4_ 0.25 g, cellulose 2 g, agar 15 g, Congo-Red 0.2 g, and gelatin 2 g, distilled water 1 l and at pH 6.8–7.2 (Gupta et al., 2011). Approximately 15 μL of an overnight culture normalized to 0.5 O.D. of the five Bcsl strains were pipetted onto the cellulose Congo-Red agar and incubated for 6 days at 30°C. Cellulytic activity was calculated by measuring the clearing zone around the bacterial colonies (Supplementary Figure S5). In addition, we mined the five Bcsl genomes for genes encoding for celluloses enzymes using RAST and the web server dbCAN.

### 2.5. DNA extraction and genome sequencing

Bcsl strains were inoculated in LB medium and incubated overnight at 30°C with shaking at 180 rpm. The overnight culture was diluted and incubated for an additional 3 h before harvesting 2 mL for genomic DNA (gDNA) isolation using the Wizard Genomic DNA Purification Kit (Promega, Madison, WI) according to the manufacturer’s instructions with slight modifications to minimize pipetting and vortex steps. gDNA yield and quality was examined using a Qubit flurometer (Thermo Fisher Scientific Inc., Waltham, MA) and a Nanodrop ND1000 spectrophotometer (NanoDrop Technologies, Wilmington, DE), and its integrity was verified by gel electrophoresis (1% agarose w/v). The gDNA was sequenced using short (Illumina MiSeq) and long (Oxford Nanopore Minion) read sequencing technologies at Genotypic Technologies, (Bengaluru, India) as described below.

A volume of 1 μg of DNA from each isolate was used for Nextra XT DNA library preparation using the manufacturers protocol (Cat#FC-131-1,024), and libraries were sequenced on an Illumina HiSeq X Ten sequencer (Illumina, San Diego, USA). In tandem, sequencing was performed on an Oxford Nanopore GridON X5 sequencing (Oxford, UK) using a SpotON flow cell R9.4 (FLO-MIN106) in a 48 h sequencing protocol. The quality of the genomes was analyzed using BUSCO (Manni et al., 2021).

### 2.6. Genome assembly and annotation

Trimmomatic software version 0.39 (Bolger et al., 2014) was used for removal of adaptors and low quality sequences using the following parameters: ILLUMINACLIP:adapters.fa:2:30:10 LEADING:3 TRAILING:3 SLIDINGWINDOW:4:15 MINLEN:36. Subsequently, Unicycler version 0.4.8 (Wick et al., 2017) was used for hybrid assembly of the trimmed Illumina paired-end and Nanopore reads using defaults parameters. The RAST server version 2 (Aziz et al., 2008; Overbeek et al., 2014; Brettin et al., 2015) was applied for the annotation of the assembled genomes using default parameters. OAT software was applied for genome comparisons of the five Bcsl strains and representative reference strains from the *Bacillus* group (*B. pumilus* NCTC10337, *B. velezensis* FZB42 and *B. subtilis* HJ5), based on OrthoANI (Average Nucleotide Identity) values (Lee et al., 2015). The online service OrthoVenn2 was used for genome wide comparisons and visualization of orthologous clusters based on protein sequences generated by RAST annotation tool (Xu et al., 2019). The pantoate-b-alanine ligase gene (*panC*) was used to identify the Bcsl strains lineage within the group (Guinebretière et al., 2008) using MEGA11 alignment of the five Bcsl strains and representative reference strains from the *B. cereus* group (*B. cereus* ATCC 14579 T, *B. thuringiensis* CIP 53137 T and *B. weihenstephanensis* INRA1). The *B. subtilis-*associated strain NYG5 was included as an outlier for rooting the tree (Supplementary Figure S3). The JSpeciesWS tool was used for calculating identity using tetra correlation of our five Bcsl strains against an updated published genome reference database (Richter et al., 2015).

The webserver antiSMASH (RRID:SCR_022060) version 5.1.2 (Blin et al., 2019) was applied for identification and annotation of secondary metabolite encoding biosynthetic gene clusters (BGC) using the default parameters. Comparative analysis of ZwA BGCs was performed and visualized with EasyFig (Sullivan et al., 2011), using the UW85 ZwA BGC as a template. The extrachromosomal plasmids were visualized using BLAST Ring Image Generator (BRIG) version 0.95 (Alikhan et al., 2011).

## 3. Results

### 3.1. Colony expansion and *in-vitro* antagonism of model phytopathogens by Bcsl strains

**Figure 1:**
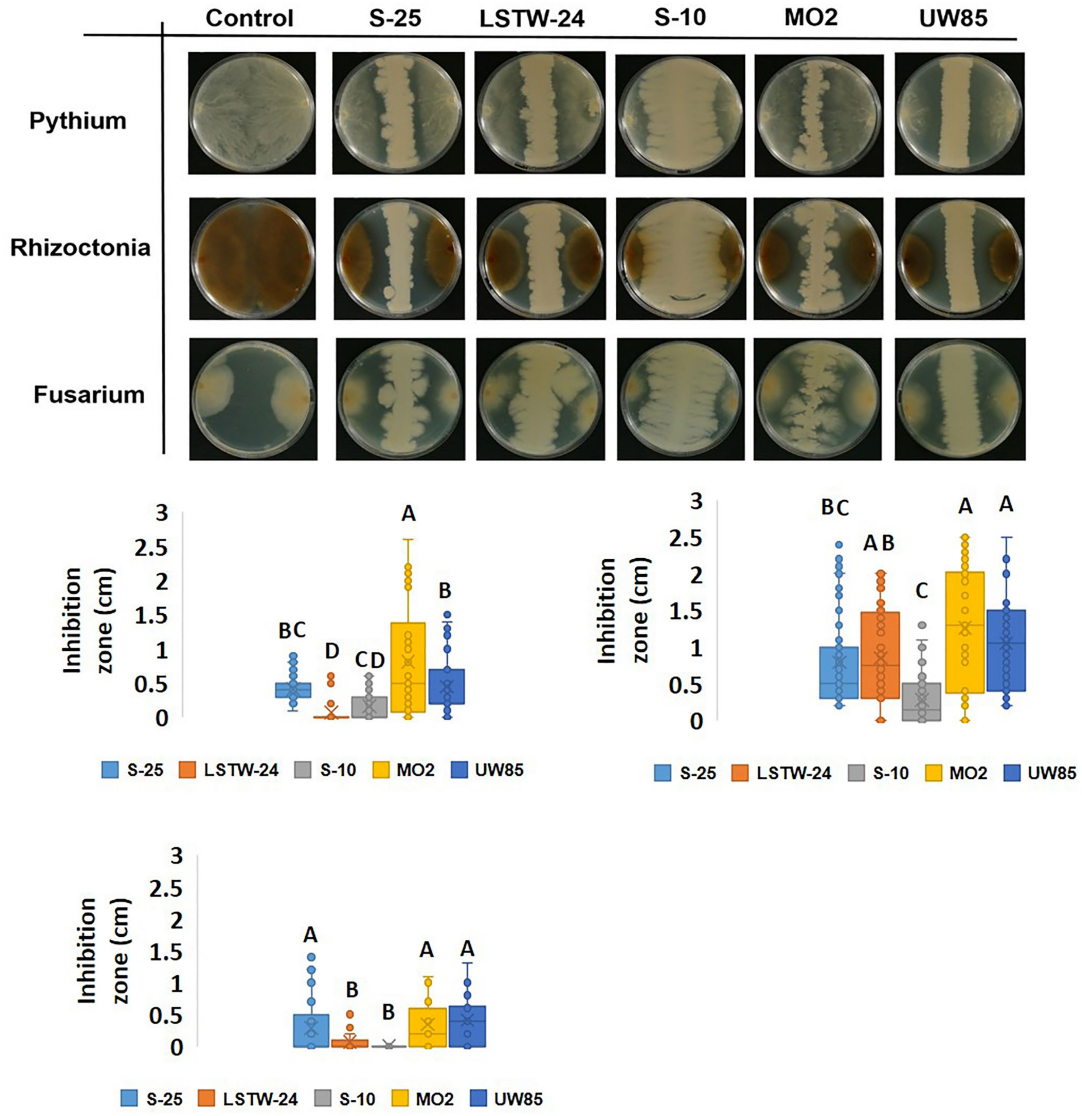
Dual culture inhibition assay of the five Bcsl strains against soilborne phytopathogens. Images of dual culture experiments (A); and inhibition zones measurements in dual cultures of 3 days old Bcsl strains with *Pythium aphanidermatum* (B); *Rhizoctonia solani* (C); and *Fusarium oxysporum* (D) on 50% LB agar at 25°C. Images are shown following 3 days of incubation for *P. aphanidermatum* and *R. solani* and 16 days of incubation for *F. oxysporum*. Box plots represent data from three independent experiments with five replicates each. The lines in the box plot represent the median while the *x* symbols represent the mean. Different letters indicate statistically significant differences (*p* < 0.05) based on the ANOVA Tukey–Kramer *post hoc* test (*α* = 0.05).

Dual culture assays revealed substantial differences in colony expansion and *in-vitro* mycelial growth inhibition by the five tested Bcsl isolates (Figure 1; Supplementary Figures S1, S2). S-10 spread rapidly and covered approximately two-thirds of the agar (resulting in substantial inhibition of *F. oxysporum*, *P. aphanidermatum* and *R. solani* mycelial expansion), whereas the other Bcsl strains grew much slower. Strain LSTW-24 did not inhibit *P. aphanidermatum* but moderately inhibited *R. solani* (~67% mycelial inhibition), while MO2 strongly inhibited *R. solani* (~92%), and moderately inhibited *P. aphanidermatum* (~54%). S-25 did not substantially inhibit *P. aphanidermatum* (~47%) and *R. solani* (~58%) mycelial growth, but caused a clear inhibition zone in dual culture assays with *P. aphanidermatum* and *R. solani* (0.5 and 0.8 cm, respectively).

### 3.2. Effect of Bcsl strain cell-free supernatant on model phytopathogen mycelial growth

**Figure 2:**
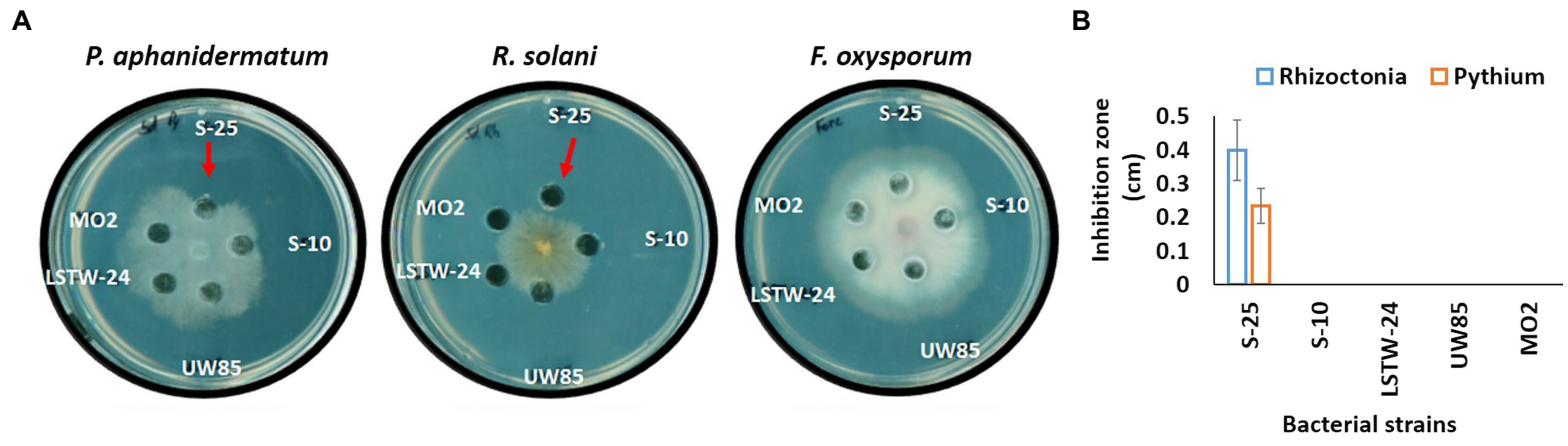
Antagonistic effect of cell-free supernatant (CFS) of the five Bcsl strains against the three model phytopathogens. Images showing antagonistic effect on mycelial growth of the three phytopathogen, *F. oxysporum*, *R. solani*, and *P. aphanidermatum* inoculated in the center of 9 cm PDA plates, mediated by CFS of 5 days old Bcsl strains grown on 50% LB medium **(A)**; Inhibition zone measurments of *R. solani* and *P. aphanidermatum* mycelia by the CFS of the five Bcsl strains **(B)**. The graph represents data from three independent experiments, and the vertical line shows standard deviations between replicates.

We tested the antagonistic effect of cell-free supernatants (CFS) of the five Bcsl strains against the three model pathogens (Figure 2). The CFS from S-25 had a clear inhibitory effect against *P. aphanidermatum* and *R. solani* mycelia (suggesting that it secretes compounds that antagonize these phytopathogens), but not against *F. oxysporum*. In contrast, the other four strains did not show a clear antagonistic phenotype against any of the screened model phytopathogens.

### 3.3. Comparative genomic analysis of Bcsl strains

Genome characteristics and sequencing metadata are summarized in Table 1. The genome sizes ranged from 5.3 to 6.3 Mbp with a mean GC-content of 35%, which is typical for members of the Bcsl group. Collectively, the genome size of Bcsl strains is larger than genomes of other *Bacillus* species used for biocontrol such as *B. Pumilus*, *B. velezensis* and *B. subtilis* (Shafi et al., 2017), whose average genome size is 3.7 Mbp, 4 Mbp and 4.1 Mbp, respectively. The total number of Open Reading Frames (ORFs) varied from 5,755 to 6,564, and the number of identified RNA genes from 107 to 138. Except for LSTW-24, all the sequenced Bcsl strains contained plasmids.

**Table 1:** Characteristics of the five Bcsl strain genomes used in this study.

Isolate name	Genome size (Mbp)	Total number of contigs^a^	Genome quality (% of completeness)	GC content (%)	N50^b^	Total open reading frames (ORFs^c^)	RNA genes	Plasmids detected
LSTW-24	5.3	46 (14)	98.7	35.2	1,355,660	5809	107	0
MO2	5.7	6 (6)	99.8	35.2	5,250,910	5755	134	5
S-25	5.9	20 (4)	99.6	35.0	5,281,513	5795	138	2
S-10	6.0	3 (3)	99.8	34.9	5,391,120	5894	134	1
UW-85	6.3	23 (6)	99.1	34.8	2,521,153	6564	129	3

a Brackets show number of contigs larger than 2,500 bp.

b Sequence length of the shortest contig at 50% of the total genome length.

c ORFs predicted by Prodigal software.

**Figure 3:**
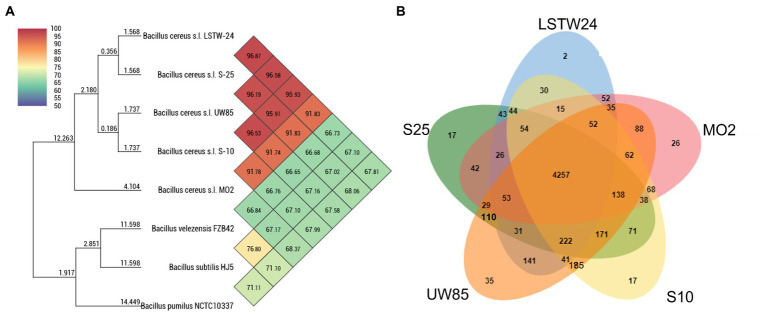
Heatmap and phylogenomic tree **(A)** showing similarity of the five-targeted Bcsl strain genomes relative to other species of *Bacillus*, based on average nucleotide identity (ANI) values, calculated using the Orthologous Average Nucleotide Identity Tool (OAT); Venn diagrams constructed using the OrthoVenn 2 online service displaying the distribution of shared and unique orthologous clusters among the five Bcsl strains **(B)**.

Average Nucleotide Identity (ANI) comparisons revealed a high level of similarity (>95%) between the Bcsl strains, with the exception of MO2 (91%, Figure 3A). Nevertheless, phylogenetic characterization of the strains based on the *pan*C housekeeping gene (involved in pantothenate biosynthesis), which has been used for phylogenetic characterization of *B. cereus* strains (Guinebretière et al., 2008), indicated that MO2 is a closely related lineage within the Bcsl group (Supplementary Figure S3). Furthermore, comparing MO2 tetra-nucleotide signatures (Tetra) to those of Bcsl strains from a large and continuously updated genome reference database using the JSpeciesWS tool, revealed a high level of identity with a correlation coefficient above 0.99. The pangenome comparisons of the five Bcsl strains identify 4,257 shared clusters out of 6,195, with 2, 17, 17, 35, and 26 clusters that were unique to LSTW-24, S-25, S-10, UW85 and MO2, respectively (Figure 3B). UW85 and MO2 accessory genes included: i) ABC transporter encoding genes, ii) genes encoding toxin and antibiotic synthesis; iii) genes linked to quorum sensing and biofilm formation, iv) genes encoding for siderophores and surfactants; v) genes encoding for carbohydrate and phosphate metabolism; and vi) genes associated with utilization of sulfur and nitrogen (Supplementary Table S1).

### 3.4. Evaluation of chitonolytic and cellulytic activity and associated metabolic genes in the Bcsl strains

**Figure 4:**
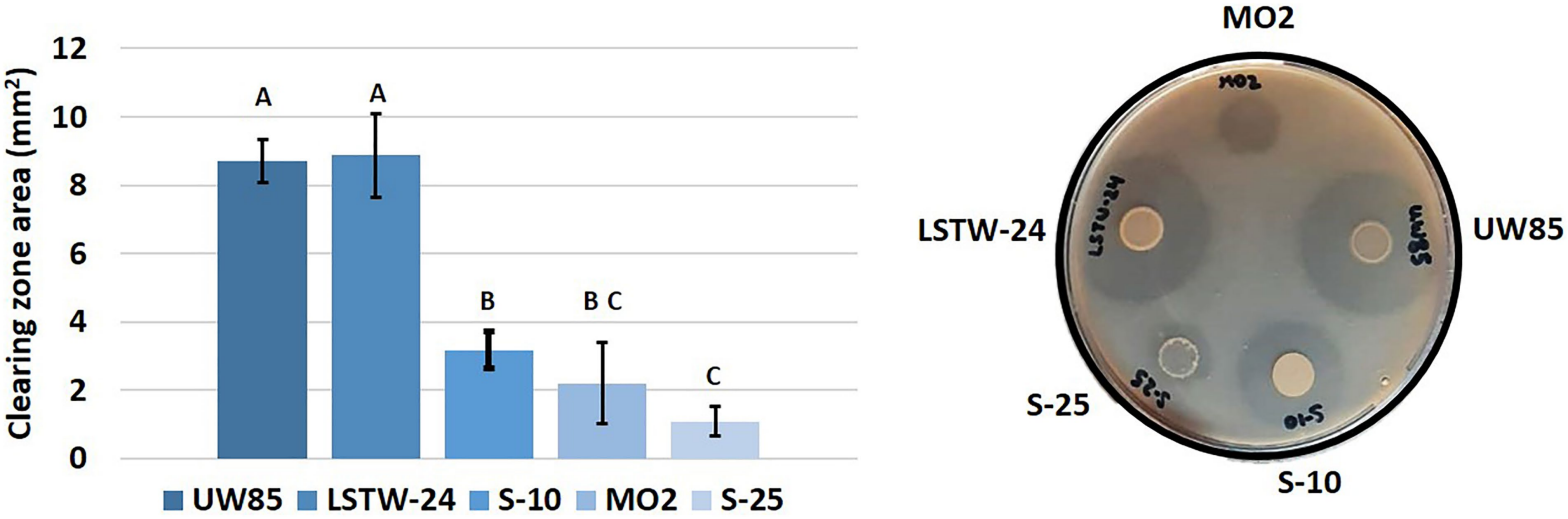
Chitinolitic activity of five Bcsl strains. Area of clearing zones indicating the different chitinolytic activity of the five Bcsl strains on M9 minimal medium containing colloidal chitin as a sole carbon source. The graph showing the mean and standard deviations of 10 independent measurements (left); and image illustrating the extracellular chitinolytic activity of the five isolates (right). Different letters indicate statistically significant differences (*p* < 0.05) based on the ANOVA Tukey-HSD test (*α* = 0.05).

We mined the five genomes for genes associated with chitin metabolism concomitant to evaluation of extracellular *in-vitro* chitinolytic activity. Two chitinase encoding genes were identified in S-25, S-10 and MO2, three in strain UW85, and four in strain LSTW-24. Furthermore, three chitin binding protein (CBP) encoding genes were detected in the five Bcsl, an additional CBP was found in S-25, LSTW-24, and MO2, whereas four additional CBP encoding genes were detected in UW85. A single endoglucanase gene potentially involved in chitin/cellulose degradation was also detected in S-25 but not in the other four Bcsl genomes. Based on blastx, each of the five isolates harbor chitinase genes belonging to subfamily A (ChiA) and subfamily B (ChiB) of the glycoside hydrolase GH18 family while only strains LSTW-24 and UW85 harbored additional chitinase genes. ChiA and ChiB were also ubiquitous in 20 additional Bcsl genomes that we screened. More than that, while additional genes associated with chitin degradation were only detected in some of the strains, the chitinases and CBPs genes found in LSTW-24 and UW85 strains were not detected in any of the other 20 analyzed Bcsl genomes (Supplementary Figure S4). The extracellular chitinolytic activity was generally proportional to the scope of genes associated with chitin degradation, with UW85 and LSTW-24 showing higher chitinolytic activity than the three other strains and S-25 showing the lowest activity (Figure 4). Similarly, we tested the five Bcsl strains for their *in vitro* cellulose-degrading capacity on cellulose-amended Congo Red agar plates (Supplementary Figure S5). Two strains (UW85 and LSTW-24) showed slightly higher cellulolytic activity than the three other strains, while MO2 showed the lowest activity. Nevertheless, despite the differences in the cellulolytic activity, we could not detect any correlation between the cellulolytic activity and the *in vitro* antagonistic activity against the oomycetes *P. aphanidermatum*, whose cell wall is composed of cellulose instead of chitin.

Screening the five Bcsl genomes did not reveal significant differences in genes encoding for cellulolytic enzymes that could explain the variances in *in-vitro* cellulolytic activity. We identified a gene encoding for 6-phospho-beta-glucosidase (EC:3.2.1.86), and the PTS transporter homologs CelC, CelB, and CelA in all of the strains. An additional gene encoding for beta-glucosidase (EC:3.2.1.21) was only identified in strains S-25, UW85 and LSTW-24. We did not identify genes encoding for endoglucanase (EC:3.2.1.4) in any of the strains except for a putative endoglucanase detected in the plasmid of the S-25 strain.

### 3.5. Annotation and comparative analysis of secondary metabolite encoding genes in Bcsl strains

**Figure 5:**
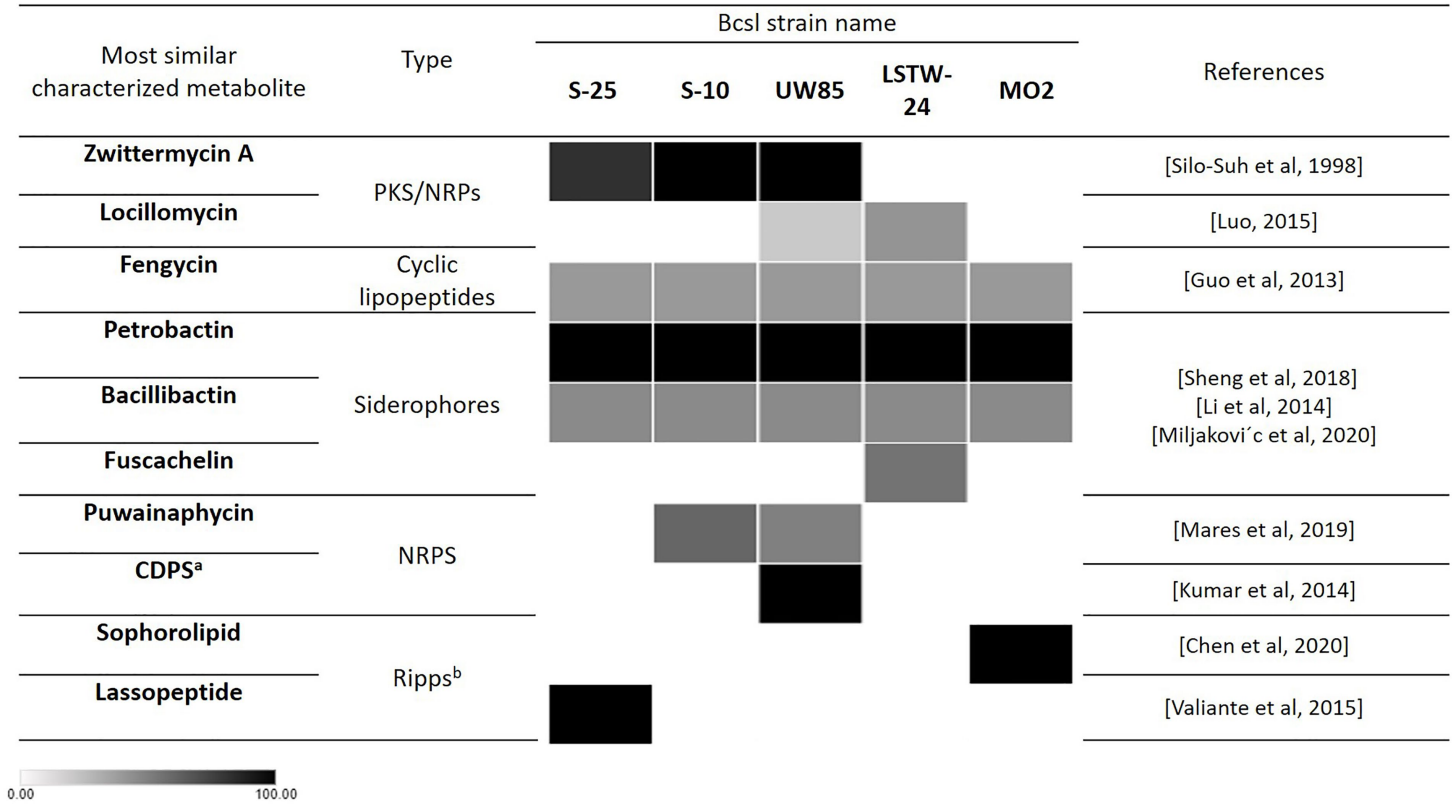
Occurrence of secondary metabolite encoding biosynthetic gene clusters with putative antifungal activity in the five analyzed Bcsl strains based on Antismash predictions. Heatmap shades represent the percentage of genes in the queried cluster that are similar to the known BGC. The darker the color the higher the percentage of similar genes in the cluster to the known BGC. ^a^CDPS: cyclic dipeptides. ^b^RiPPs: Ribosomally synthesized and post-translationally modified peptides.

The Bcsl genomes were screened for documented and potentially novel secondary metabolite encoding BGCs using Antismash (Medema et al., 2011), which ranks gene clusters by similarity to a queried gene cluster of known function, presenting the percentage of genes in the queried cluster that show similarity to the known BGC (Figure 5). All five genomes harbored BGCs encoding for the catecholate siderophores petrobactin and bacillibactin (100 and 46% similarity, respectively) (Lee et al., 2011). LSTW-24 harbored an additional BGC with 55% similarity to the siderophore fuscachelin. All genomes harbored a BGC with 40% similarity to fengycin, a biologically active lipopeptide produced by several *B. subtilis* strains known to antagonize filamentous fungi (Deleu et al., 2008). A BGC similar to Locillomycin was detected in UW85 (21%) and two such clusters (28 and 42% identity, respectively) were identified in LSTW-24. Another BGC similar to the lipopeptide Puwainaphycin was detected in UW85 (50% identity) and S-10 (60% identity). Each of the five genomes contained unique secondary metabolite encoding BGCs. For example, S-25 carried a lassopeptide-encoding BGC, MO2 harbored a BGC encoding for a sophorolipid (100% identity), a group of amphiphilic biosurfactants, and UW85 carried a BGC predicted to encode for an unknown cyclodipeptide. In addition, S-25 and S-10 harbored BGCs putatively encoding for the hybrid NRPS/PKS metabolite ZwA (81 and 100% similarity, respectively), which was first characterized in UW85 (Silo-Suh et al., 1994). This metabolite has been extensively explored in UW85 and other prospective Bcsl biocontrol strains, due to its antagonistic effect against oomycetes and other soilborne pathogens (Raffel et al., 1996; Silo-Suh et al., 1998; Broderick et al., 2000; Zhao et al., 2007).

### 3.6. Comparative analysis of ZmA harboring mega-plasmids

**Figure 6:**
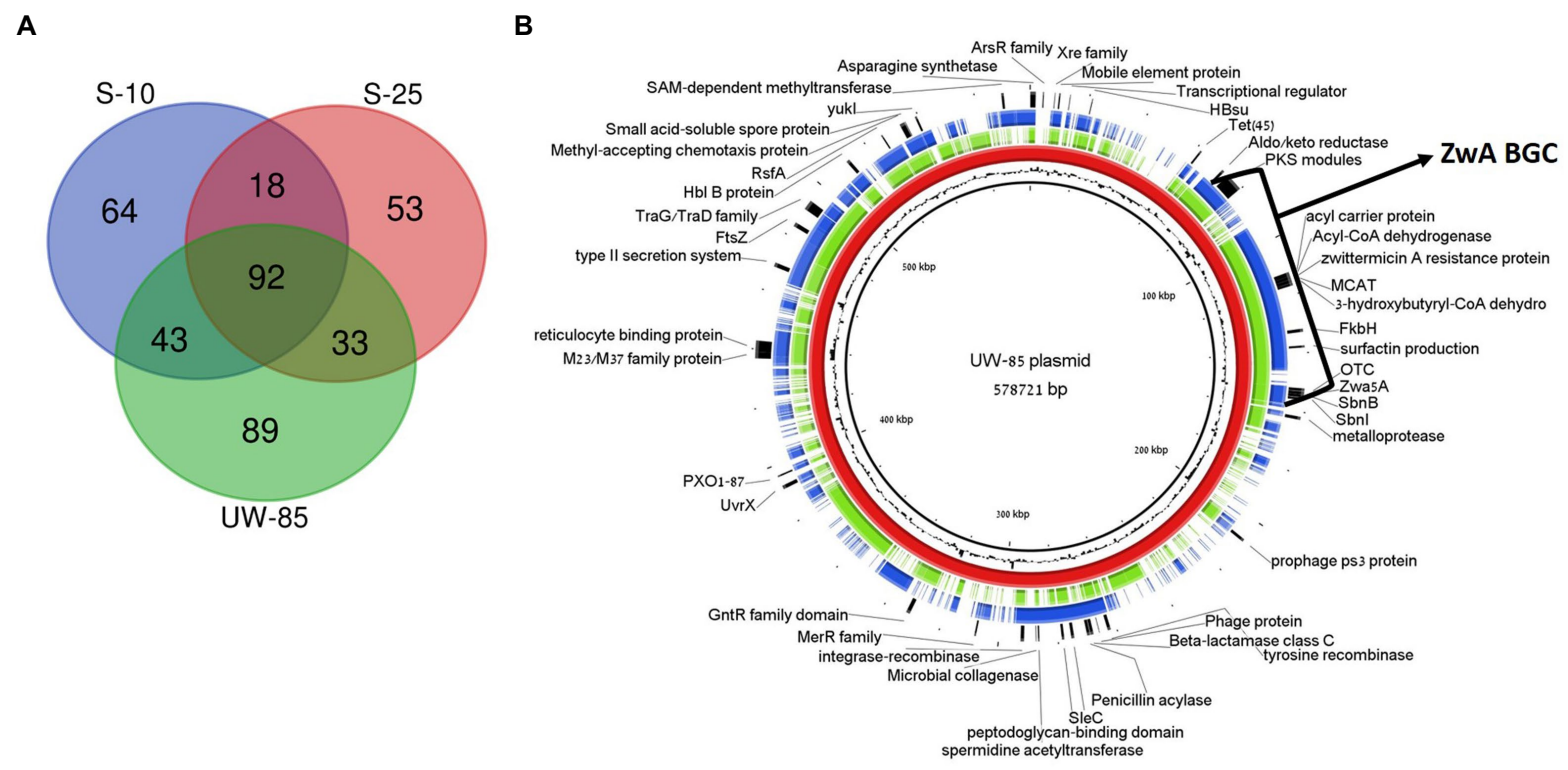
Comparative analysis of plasmids containing ZwA BGC homologues in *Bacillus cereus* spp. UW85, S-10 and S-25. **(A)** Venn-diagram showing similar and unique RAST annotated genes in the three plasmids. **(B)** Genetic map of the S-10 (green) and S-25 (blue) plasmids aligned against the UW85 reference plasmid (red) using the BRIG software package. The annotations of relevant encoded proteins from three sequenced plasmids appear in the outer black ring, and the GC content of the reference plasmid is displayed between the inner black and red rings.

We compared the mega-plasmid harboring the ZmA encoding BGC in UW85 to its two homologs in S-25 and S-10. The UW85, S-25 and S-10 mega-plasmids were 578,721, 436,050, and 588,156 bp, encoding for 636, 404 and 530 genes, respectively. The GC content of all of the plasmids was approximately 32%, which was slightly lower than that of the chromosomes. A total of 257, 196 and 217 genes, respectively, were functionally annotated by RAST, almost half (92) of whom were shared between the three strains (Figure 6A).

Approximately 12% of the annotated mega-plasmid genes (16% of shared genes) were predicted to be part of the ZwA BGC (Figure 6B). Common annotated genes found on the plasmid that were not part of the ZmA BGC included genes encoding for putative virulence factors and pathogenesis factors (i.e., microbial collagenase, hemolysin B, reticulocyte binding protein, and type II secretion systems), ABC transporters, tetracycline and β-lactam resistance and quorum sensing and chemotaxis mechanisms (Figure 6B). In addition, 89, 53, and 64 of the annotated genes were unique in the UW85, S-25, and S-10 mega-plasmids, respectively, representing over 50% of the unique genes reported above. The UW85 mega-plasmid contained nine genes encoding for ABC transporters that were absent in the other two strains (Supplementary Table S2). The S-25 mega-plasmid contained genes encoding for chitin binding proteins and a predicted endoglucanase, potentially involved in antifungal activity, which were absent in the other two strains. (Supplementary Figure S6; Supplementary Table S2).

### 3.7. Comparative analysis of ZmA BGCs

**Figure 7:**
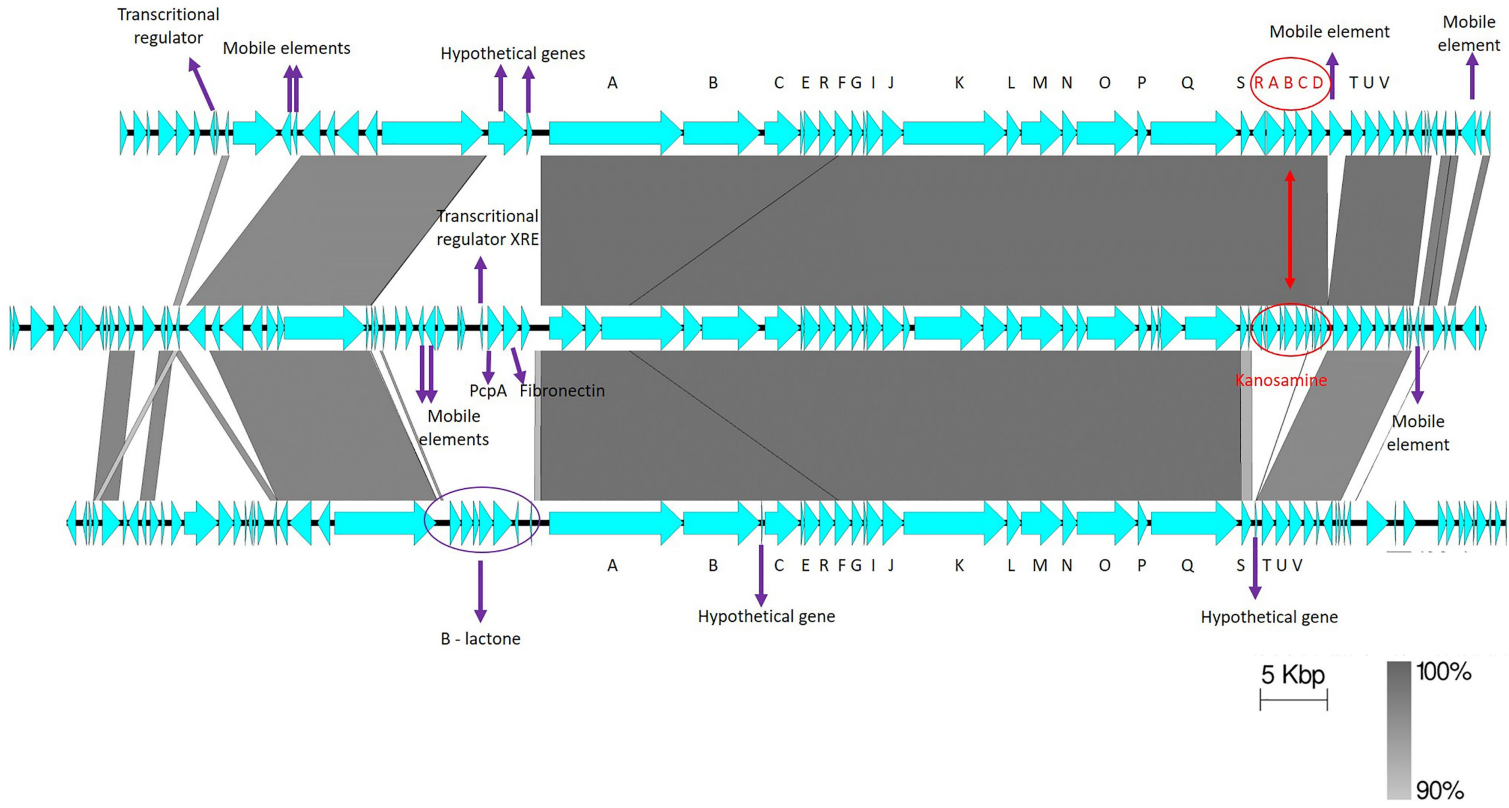
Synteny of ZmA homolog BGCs. The reference ZwA BGC of UW-85 (middle), is flanked by S-10 (top), and S-25 (bottom) homologues. The annotations of relevant encoded proteins from three BGCs are indicated by arrows. ZmA BGC genes are shown in black capital letters A–V, and kanosamine encoding genes in red capital letters. The figure was generated using Easyfig software with a cut-off of 90% gene identity.

Gene synteny comparisons between UW85 ZmA BGCs and homologs from S-25 and S-10 revealed high similarity between the clusters (Figure 7), except for a group of genes predicted to encode for the antibiotic kanosamine situated within the ZmA BGCs of UW85 and S-10, but absent in the S-25 BGC. In contrast, the ZmA encoding BGC of S-25 contained genes predicted to encode for a β-lactone containing protease inhibitor located proximal to the ZmaA BGC, which was absent in the other two strains. In addition, two hypothetical protein-encoding genes were identified in the ZmA-encoding BGC of S-25, whereas a mobile element sequence proximal to the kanosamine encoding gene was detected in S-10.

## 4. Discussion

The five analyzed Bcsl strains described in this study displayed unique *in-vitro* antagonistic profiles against fungal and fungal-like (i.e.*,* Ascomycota, Basidiomycota, and Oomycetes) plant pathogens as previously demonstrated for other Bcsl strains (Silo-Suh et al., 1994; Xu et al., 2014; Kumar et al., 2014b). We therefore mined their genomes to pinpoint genes and gene clusters encoding potential antifungal factors associated with the observed antagonism and performed comparative genomic analysis to identify specific factors that might explain differences in their *in-vitro* antagonistic phenotypes.

Collectively, more unique genes were detected in UW85 and MO2 relative to the three other Bcsl strains. This increased diversity may be explained by the fact that they were isolated from plant roots in contrast to the other three strains, which were isolated from bulk soil, although it is sometimes difficult to differentiate between these two niches. Root ecosystems are considered to be more competitive than bulk soil, necessitating genes encoding specific characteristics to ensure survival (Lugtenberg and Dekkers, 1999; Zhang et al., 2016; Ling et al., 2022). An example are genes encoding for transporters which facilitate the uptake of root-associated nutrients and essential molecules, or remove antibiotics or other toxic compounds produced by competitors in the root environment from the cell (Rees et al., 2009). The involvement of ABC transporters in biocontrol and rhizosphere competence is well established. For example, the ABC transporter abcG5 in the fungal biocontrol agent *Clonostachys rosea* was recently found to be essential for biocontrol activity against *F. graminearium*-facilitated foot rot disease (Dubey et al., 2014), and a gene encoding for a putative ABC transporter from *Erwinia chrysanthemi* was found to play a role in *in planta* fitness of the bacterium (Llama-Palacios et al., 2002). It is possible that the presence of additional ABC transporter encoding genes on the UW85 ZwA mega-plasmid may contributes to UW85 capacity to colonize and persist in the rhizosphere and therefore potentially enhances its biocontrol activity.

The presence of unique genes involved in carbohydrate and phosphate metabolism in the UW85 and MO2 genomes may also contribute to the capacity of these strains to survive in the copiotrophic plant root ecosystem. This is supported by a comparative genomic analysis of plant associated vs. non-plant-associated *B. amyloliquefaciens* and *B. subtilis* strains, which showed that plant-associated strains possess additional genes involved in utilizing plant-derived substrates, seemingly acquired through horizontal gene transfer (HGT; Zhang et al., 2016). UW85 and MO2 also contained more transposase genes, which may indicate higher occurrence of HGT events that can facilitate the acquisition of genes associated with environmental adaptation (Aminov, 2011; Raymond and Bonsall, 2013).

### 4.1. Linking chitinolytic activity and chitin degrading genes in Bcsl strains

Bacterial chitinases that degrade α-1,3-glucans, and β-1,3-glucans (the major components of fungal cell walls) can play a fundamental role in fungal antagonism by biocontrol strains (Swiontek Brzezinska et al., 2014). For example, *chiA* encoded chitinases in *Serratia marcescens* and *S. plymuthica* were linked to biological control of plant diseases caused by phytopathogenic fungi (Chernin et al., 1997; Downing and Thomson, 2000), and *B. thuringiensis* isolates from tomato roots only exhibited *in-vitro* antifungal activity against *Verticillium* spp. when harboring one or two putative chitinases (Hollensteiner et al., 2016; Veliz et al., 2017). Interestingly, the chitinolytic activity of the five Bcsl isolates investigated in this study correlated to their genetic potential, with higher activity documented in UW85 (which harbored almost twice as many CBPs as the other strains) and LSTW-24 (which contained more chitinases than the other strains). Previous reports have explored the genetic context of chitinase genes (Yan and Fong, 2015), the association between molecular structure, substrate specificity, the catalytic mechanisms that facilitate chitinase activity (Hamid et al., 2013), and the synergistic activity of different chitinases (Suzuki et al., 2002). The linkage between the quantity of chitin degrading genes and the chitinolytic activity of strains UW85 and LSTW-24 documented here is supported by previous studies suggesting that synergistic interactions between CBP and chitinases enhance the capacity of biocontrol agents to metabolize chitin (Purushotham et al., 2012; Manjeet et al., 2013; Veliz et al., 2017).

S-25 harbors a unique gene encoding a probable endoglucanase on its mega-plasmid. Previous studies demonstrated that endoglucanase activity antagonized *P. aphanidermatum* (Natarajan et al., 2013) and reduced disease incidence by *Pythium* on cucumber seedlings (Chet et al., 1998). Other studies reported that a b-1,3-glucanase facilitated morphological changes and growth inhibition of *R. solani* and *Fusarium* sp. (Benitez et al., 1998; Bhat, 2000). In view of these findings, we suggest further examination of this endoglucanase gene, and specifically examination of its involvement of S-25 antagonism of *P. aphanidermatum* and *R. solani*. The proximity between this endoglucanase gene and three CBPs genes may imply combined antifungal activity of these genes.

While our analyses revealed strong correlations between chitinase activity and the abundance of chitinase encoding genes, there was no correlation between extracellular chitinolitic activity, and the antifungal activity of the CFS, suggesting that either particular chitinases may be more active against specific fungi or that additional antagonistic mechanisms are also required.

### 4.2. Presence of secondary metabolite encoding BGCs in Bcsl strains with putative antifungal activity

Secondary metabolites play a critical role in the antagonism of phytopathogens by bacterial biocontrol agents (Vizcaino et al., 2014) and many secondary metabolites with antifungal activity have been detected in *Bacillus spp.* (Shahid et al., 2021). We therefore screened the five Bcsl genomes for secondary metabolite encoding genes and performed comparative genomic analyses to uncover the genetic basis of the observed differences in their *in-vitro* antagonistic capacity.

Shared BGCs, common to all of the five analyzed strains and having putative antifungal activity, included clusters encoding for catecholate siderophores similar to petrobactin and bacillibactin, which were previously shown to play vital roles in the antagonistic capacity of various bacterial biocontrol agents against phytopathogens (Prema and Selvarani, 2012; Li et al., 2014; Patil et al., 2014; Miljakovic et al., 2020; Sheng et al., 2020). Another shared genes cluster is similar to the antifungal lipopeptide fengycin, which was shown to inhibit *R. solani* (Guo et al., 2013) and other fungal pathogens (Ongena et al., 2005; Deleu et al., 2008; Kulimushi et al., 2017). In contrast, several unique clusters, which may contribute to the specific antifungal activity of each Bcsl strain, were also detected. These include a BGC, only detected in the UW85 and LSTW-24 genomes, having similarity (21%–42%) for the NRPs-PKs hybrid locillomycin, reported to antagonize *R. solani* and *F. oxysporum* (Luo, 2015) and a BGC found in S-10 and UW85 genomes with similarity (50–60%) to a puwainaphycin lipopeptide, which was previously shown to possess antifungal activity against members of the Ascomycota phylum (Mares et al., 2019; Hajek et al., 2021). Interestingly, in our research we did not observe inhibition of *F. oxysporum* which represent the Ascomycetes soilborne pathogen, but this predicted gene cluster similar to puwainaphycin need to be further explored for its role in the inhibition activity of these two strains against *R. solani* and *P. aphanidermatum*. Sophorolipid, only detected in MO2, is an extracellular bio-surfactant reported to inhibit mycelial growth of *R. solani* (64.3%) and *P. ultimum* (95%; de OCaretta et al., 2021), in addition to other fungal phytopathogens in both *in vitro* and *in planta* experiments (Yoo et al., 2005; Sen et al., 2017; Haque et al., 2019; Chen et al., 2020; de OCaretta et al., 2021). The presence of this antifungal encoding BGC in MO2 genome may contribute to its observed antagonistic activity against *R. solani* and *P. aphanidermatum*. In addition, S-25 carried unique BGC encoding for a lassopeptide, a group of natural products previously shown to have therapeutic effects on fungal infections, as demonstrated for the class I lassopeptide humidimycin from *Streptomyces humidus*. This compound expedited activity of fungal cell wall inhibitors that antagonized *Candida albicans* and *Aspergillus fumigatus* (Valiante et al., 2015). The different profiles of secondary metabolite encoding genes between the five Bcsl strains may partially explain their different antifungal phenotypes against the three soilborne pathogens, but additional work is required to clarify their exact role in the observed antagonistic activity.

### 4.3. Comparative analysis of ZwA BGC and the ZwA harboring plasmid

S-25 and S-10 harbored homologs of the well-established antifungal hybrid NRP/PK zwittwrmicin A, a linear aminopolyol antibiotic originally isolated from UW85 (Kevany et al., 2009). ZwA has been previously shown to supress alfalfa seedlings damping off caused by the oomycete *P. medicaginis* (Handelsman et al., 1990), it has been indicated in inhibition of other fungal and bacterial growth (Silo-Suh et al., 1998) and has been shown to enhance insecticidal activity of Cry toxins in *B. thuringiensis* (Broderick et al., 2003). The expression and activity of ZwA might likely be different in the three strains, because S-25 BGC lacked the five flanking kab (kabA-kabD; kabR) genes that encode for the antifungal element kanosamine (Janiak and Milewski, 2001), and S-25 contained a flanking gene encoding for a beta-lactone containing protease inhibitor that was absent in S-10 and UW85. The contribution of flanking genes to zwittermicin activity is supported by Luo et al., who identified zmaWXY downstream of ZmA that functioned as a resistance conferring in addition to the previously characterized zmaR gene, and was found to increase the yield of ZmA (Luo et al., 2011).

The antagonistic capacity of ZwA, and the documented role of plasmids in environmental adaptation (Heuer and Smalla, 2012; Adams et al., 2014; Patino-Navarrete and Sanchis, 2016; Lechuga et al., 2020), led us to further explore the composition of the ZmA-harboring plasmids. We detected several genes on the three plasmids with putative roles in environmental adaptation. These include as methyl-accepting chemotaxis protein (MCP) which has been previously shown to be involved in chemical sensing (Salah Ud-Din and Roujeinikova, 2017), biofilm formation (Hickman et al., 2005) and production of toxins (Harkey et al., 1994), and the two-component system sensor histidine kinase. These sensory mechanisms and response regulators are believed to enhance fitness in bacteria from unstable and low nutrient environments with multiple interactions like soil (Ashby, 2004). Each of the plasmids had its own set of unique genes, which may also have an impact on the biocontrol potential of the isolate. These included increased abundance of ABC transporters on the UW85 mega-plasmid, and the presence of chitin (CBPs) and cellulose (endoglucanase) metabolizing enzyme encoding genes gathered in S-25 mega-plasmid.

Collectively, we identified both common and unique BGCs encoding for metabolites with putative antifungal activity in the five Bcsl strains, as well as chitinases with potential antifungal activity. Nonetheless, the potential link between these factors and the observed *in-vitro* antifungal capacity of the Bcsl strains that harbor them, needs to be experimentally validated by knockout (Guo et al., 2013) and/or heterologous expression (Luo et al., 2011), demonstrated for fengycin and zwittermicin, respectively. Experiments should also be conducted to determine the expression of candidate genes and BGCs following exposure to pathogens, or under different environmental conditions, as previously described for lipopeptides and bacilibactin (Li et al., 2014).

In summary, comparative genomics provided important insights into similarities and differences of mechanisms potentially linked to the antifungal activity of the five strains. Although there are several potential candidates, we were not able to specifically link genotypes to phenotypes, or pinpoint genetic factors that explain the elevated activity of the S-25 cell-free supernatant relative to the other strains. Future studies will need to follow up on these candidates in order to validate their antifungal capacity.

## Data availability statement

The datasets presented in this study can be found in online repositories. The names of the repository/repositories and accession number (s) can be found at: https://www.ncbi.nlm.nih.gov/, JAKJPR000000000; https://www.ncbi.nlm.nih.gov/, JAKJPS000000000 https://www.ncbi.nlm.nih.gov/, JAKJPT000000000; https://www.ncbi.nlm.nih.gov/, JAKJPU000000000; https://www.ncbi.nlm.nih.gov/, CP091444; https://www.ncbi.nlm.nih.gov/, PRJNA784166.

## Author contributions

MM: conducted experiments, data and bioinformatics analyses, wrote manuscript. CG: bioinformatics analysis. NS: genome assembly. DM: project idea, funding acquisition. EB: supervision. EC: experimental design, supervision, funding acquisition, writing and revisions. OF: participating in experimental design, supervision, funding acquisition, writing and revisions. RJ: isolation and conducting of the initial in-vitro antifungal analysis on three of the five bacteria investigated in the manuscript. All authors contributed to the article and approved the submitted version.

## Funding

This research was funded by the Israeli Chief Scientist of the Ministry of Agriculture and Rural Development (Grant no. 20-13-0027). In addition, MM was supported by scholarships from the Israeli Ministry of Innovation, Science and Technology, and the Avi Greinstein fellowship.

## Conflict of interest

The authors declare that the research was conducted in the absence of any commercial or financial relationships that could be construed as a potential conflict of interest.

## Publisher’s note

All claims expressed in this article are solely those of the authors and do not necessarily represent those of their affiliated organizations, or those of the publisher, the editors and the reviewers. Any product that may be evaluated in this article, or claim that may be made by its manufacturer, is not guaranteed or endorsed by the publisher.
