# Corrigendum: Comparative genomics of *Bacillus cereus sensu lato* spp. biocontrol strains in correlation to *in-vitro* phenotypes and plant pathogen antagonistic capacity

**DOI:** 10.3389/fmicb.2023.1271554

**Published:** 2023-10-23

**Authors:** Maya Moshe, Chhedi Lal Gupta, Rakeshkumar Manojkumar Jain, Noa Sela, Dror Minz, Ehud Banin, Omer Frenkel, Eddie Cytryn

**Affiliations:** ^1^Institute of Soil, Water and Environmental Sciences, Agricultural Research Organization, Rishon-Lezion, Israel; ^2^Institute of Plant Pathology and Weed Research, Agricultural Research Organization, Rishon-Lezion, Israel; ^3^The Mina and Everard Goodman Faculty of Life Sciences, Bar-Ilan University, Ramat Gan, Israel

**Keywords:** biocontrol agent, chitinase, comparative genomics, phytopathogen, secondary metabolites, zwittermicin

In the published article, there was an error in the author list, and author Rakeshkumar Manojkumar Jain was erroneously excluded. The corrected author list appears below.

Maya Moshe, Chhedi Lal Gupta, Rakeshkumar Manojkumar Jain^1^, Noa Sela, Dror Minz, Ehud Banin, Omer Frenkel, and Eddie Cytryn.

^1^Institute of Soil, Water and Environmental Sciences, Agricultural Research Organization, Rishon-Lezion, Israel

The correct author contributions section appears below:

## Author contributions

MM: conducted experiments, data and bioinformatics analyses, and wrote manuscript. CG: bioinformatics analysis. NS: genome assembly. DM: project idea and funding acquisition. EB: supervision. EC: experimental design, supervision, funding acquisition, writing, and revisions. OF: participating in experimental design, supervision, funding acquisition, writing, and revisions. RJ: isolation and conducting of the initial *in-vitro* antifungal analysis on three of the five bacteria investigated in the manuscript. All authors contributed to the article and approved the submitted version.

The authors apologize for this error and state that this does not change the scientific conclusions of the article in any way. The original article has been updated.

