# Supplementary material for: Comparative genomics of Bacillus cereus sensu lato spp. biocontrol strains in correlation to in-vitro phenotypes and plant pathogen antagonistic capacity
Source: Front Microbiol. 2023 Feb 9;14:996287. doi: 10.3389/fmicb.2023.996287 (PMC9947482; doi:10.3389/fmicb.2023.996287)
Supplement: Supplementary file 1 [file Data_Sheet_1.pdf]

Supplementary Figure S1 JPEG

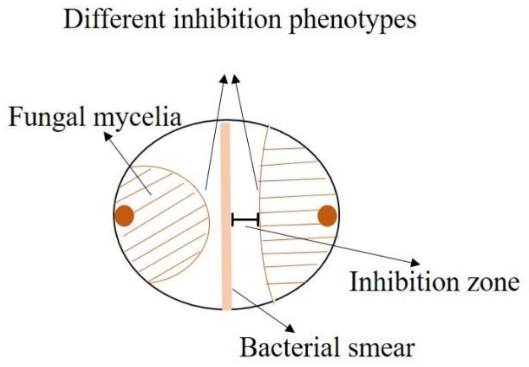

Supplementary Figure S1. Schematic diagram of *in vitro* antagonistic activity test demonstrating the different modes of action.

Supplementary Figure S2 JPEG

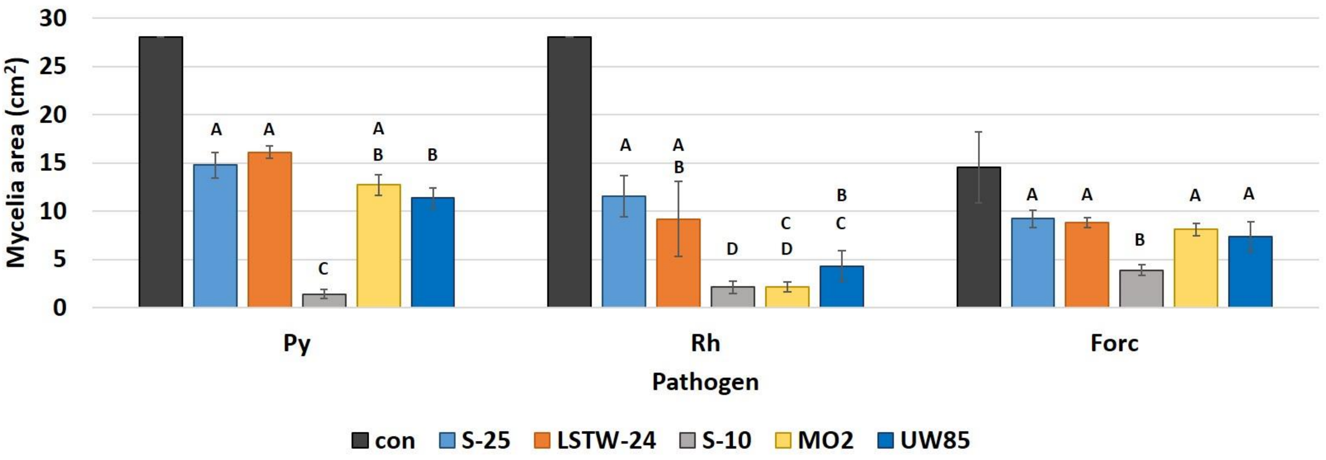

Supplementary Figure S2. Mycelial growth inhibition in a dual culture assay of five Bcsl strains against soilborne phytopathogens. Calculated mycelial area (as area of an ellipse = π*R1*R2) of the soilborne pathogens *Pythium aphanidermatum* (Py); *Rhizoctonia solani* (Rh); and *Fusarium oxysporum* (Forc) following three days of incubation for Py and Rh and sixteen days of incubation for Forc with and without each of the five Bcsl strains. The results represent data from three independent experiments with five replicates each. Different letters indicate statistically significant differences (P< 0.05) based on the ANOVA Tukey-Kramer post hoc test (α=0.05).

Supplementary Figure S3 JPEG

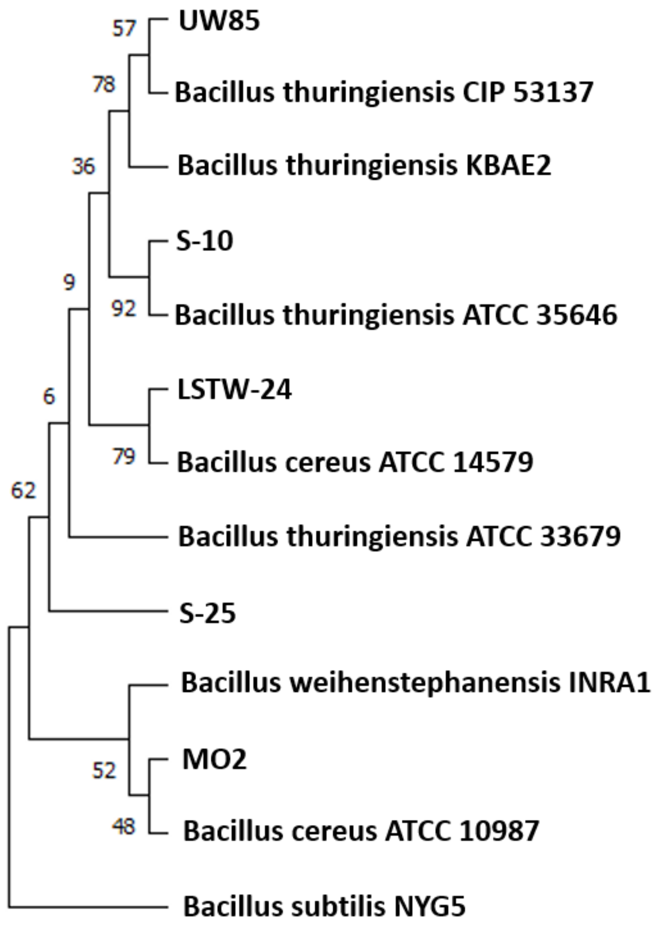

Supplementary Figure S3. Phylogenetic lineage assignment of the five Bcsl strains within the *B cereus s.l.* group based on *panC* sequences analysis.

Neighbour-joining phylogeny constracted using MEGA11 alingment of *panC* sequences derived from the five Bcsl isolates, representative strains of a different species in the *B. cereus* group. (B. cereus ATCC 14579T , *B. thuringiensis* CIP 53137T and *B. weihenstephanensis* INRA1) and outgroup genome of another isolate (NYG5), belonging to the *Bacillus subtilis* species was used to root the phylogeny. The value at each node represents the bootstrap value (1,000 replicates).

Supplementary Figure S4 JPEG

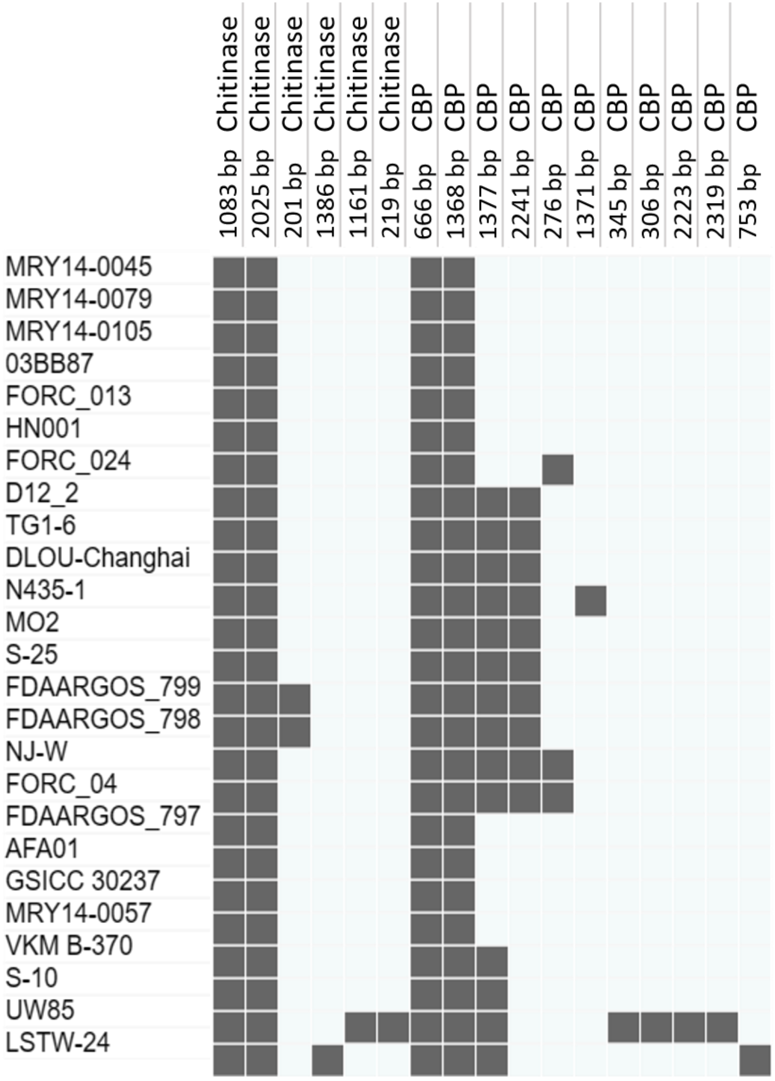

Supplementary Figure S4. Variability of chitin metabolism genes in the Bcsl group. Comparison of chitin metabolism-encoding genes from the five Bcsl genomes along with twenty randomly selected Bcsl genomes from the NCBI database. Genes were annotated using the RAST annotation tool. The Heatmap was generated using Morpheus, https://software.broadinstitute.org/morpheus.

Supplementary Figure S5 JPEG

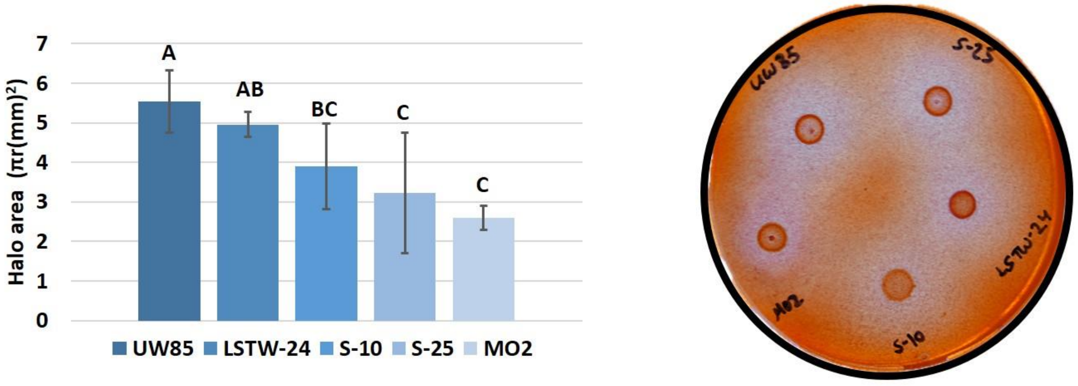

Supplementary Figure S5: Cellulolytic activity of five Bcsl strains. Area of clearing zones showing the mean and standard deviations of 7 independent measurements (left). Different letters indicate statistically significant differences (P< 0.05) based on the ANOVA Tukey-HSD test (α=0.05); and image illustrating the extracellular cellulolytic activity of the five isolates on cellulose Congo Red agar plates after six days of incubation (right). The formation of clearing zone around the colonies confirms the secretion of extracellular cellulose.

Supplementary Figure S6 JPEG

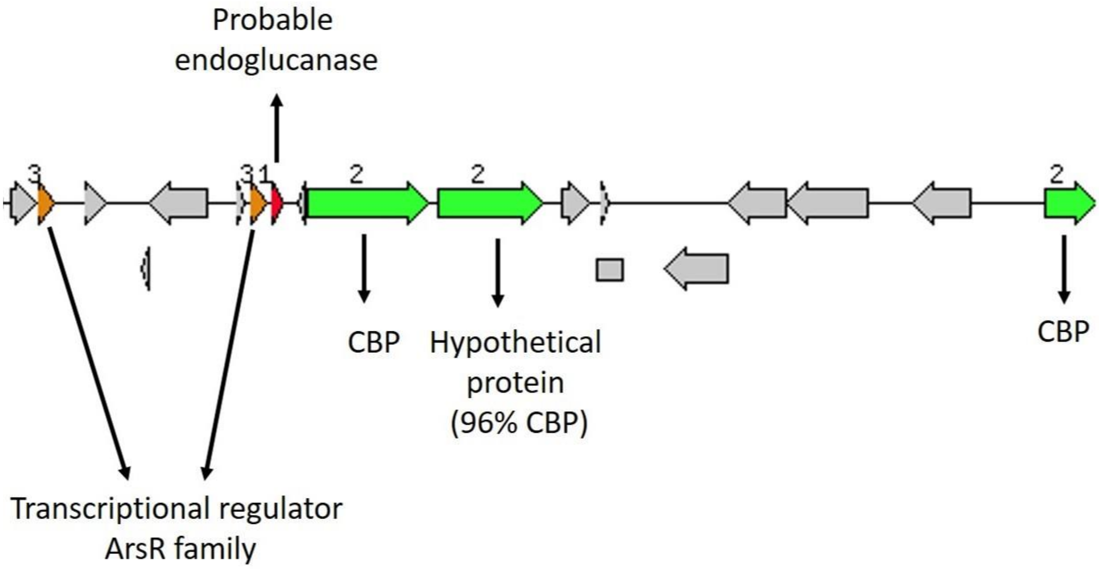

Supplementary Figure S6. Region of antifungal related genes on the S-25 mega-plasmid. Genes with similar sequences are grouped with the same number and color.
