# Supplementary material for: Comparative genomics of Bacillus cereus sensu lato spp. biocontrol strains in correlation to in-vitro phenotypes and plant pathogen antagonistic capacity
Source: Front Microbiol. 2023 Feb 9;14:996287. doi: 10.3389/fmicb.2023.996287 (PMC9947482; doi:10.3389/fmicb.2023.996287)
Supplement: Supplementary file 2 [file Data_Sheet_2.PDF]

Supplementary Table S1: unique genes detected by RAST among the five Bcsl isolates

isolates / Genes category	S-25	S-10	LSTW-24	UW85	MO2
**ABC transporter and antimicrobial resistance genes**	Oligopeptide ABC transporter,OppA	Manganese ABC transporter, SitA	ABC-type multidrug transport system, ATPase component	ABC transporter membrane-spanning permease - macrolide efflux	Bacteriocin/lantibiotic efflux ABC transporter, permease
	Purine efflux pump PbuE	ABC-type multidrug/protein/lipid transport system, ATPase component	ABC transporter, MDR family protein	ABC transporter-coupled two-component system, ATP-binding protein	Dipeptide ABC transporter, permease protein DppC
	Transmembrane transport protein MmpL family	Manganese ABC transporter, SitD	ABC transporter, MDR family protein	ABC transporter-coupled two-component system, signal transduction histidine kinase	oligopeptide ABC transporter, ATP-binding protein, putative
	Two-component response regulator YvcP	Manganese ABC transporter, SitB	5-methylthioribose ABC transporter, substrate-binding protein	Oligopeptide ABC transporter, ATP-binding protein	Teichoic acid export ATP-binding protein TagH
		Drug resistance transporter, EmrB/QacA subfamily	5-methylthioribose ABC transporter, ATP-binding protein	ABC transporter-like sensor linked histidine kinase	Membrane protein involved in the export of O-antigen, teichoic acid lipoteichoic acids
		Oligopeptide transport ATP-binding protein OppF	Heterodimeric efflux ABC transporter, permease	ABC transporter, permease	Teichoic acid translocation permease protein TagG

		Subtilin transport ATP-binding protein spaT	5-methylthioribose ABC transporter, permease protein	ABC transporter-coupled two-component system, LuxR family response regulator	Homoserine/threonine efflux protein
		Oligopeptide transport system permease protein OppB	Zwittermicin A resistance protein ZmaR	ABC transporter-coupled two-component system, fused permease protein	Multidrug resistance protein B
			GNAT family acetyltransferase BA2847	Major facilitator superfamily MFS_1 (Major Facilitator Superfamily (MFS) transporters play an important role in multidrug resistance in fungi.)	blasticidin S deaminase, putative
			penicillin-binding protein, putative	Cobalt-zinc-cadmium resistance protein CzcA; Cation efflux system protein CusA	Acetyltransferase, GNAT family
				Membrane translocator	Uncharacterized transcriptional regulator YdeS, TetR family
				putative transport permease yfiM	putative mercury resistance protein
				acetyltransferase, GNAT family	Probable poly(beta-D-mannuronate) O-acetylase
				Histone acetyltransferase HPA2 and related acetyltransferases	Uncharacterized protein YvaD
				Haloacid dehalogenase-like hydrolase	General secretion pathway protein E

				Pleiotropic drug resistance protein yfiN1	Lactoylglutathione lyase
				Methylenomycin A resistance protein	TSPc, tail specific protease
				Histone acetyltransferase HPA2 and related acetyltransferases	CAAX amino terminal protease family family
				Penicillin-binding protein	penicillin-binding protein
				Putative EsaC protein analog (Listeria type 3)	
**Toxins and Antibiotic synthesis genes**		Lanthionine biosynthesis protein LanB	HlyC domain protein	SCIFF radical SAM maturase	tcdA-E operon negative regulator
		Macrolide 2'-phosphotransferase, putative		bacteriocin immunity protein	Coenzyme F420-dependent N5,N10-methylene tetrahydromethanopterin reductase and related flavin-dependent oxidoreductases; sulfonate monooxygenase
		Lanthionine biosynthesis cyclase LanC		Bacitracin synthetase 3 (BA3)	btrG family protein
		lichenicidin prepeptide		Surfactin synthetase subunit 1	TldD protein, part of TldE/TldD proteolytic complex
					haemolytic enterotoxin
					Lmo0066 homolog within ESAT-6 gene cluster,

					similarity to ADP-ribosylating toxins
**Pathogenesis related genes and virulence factors**	collagen triple helix repeat domain protein		pathogenesis related protein	Choline binding protein PcpA	GtrA family protein
	Genomic island nu Sa beta2		Glycoside hydrolase, family 25	Bacillosamine/Legionaminic acid biosynthesis aminotransferase PglE	ESAT-6-secreted WXG100 domain protein EsxV (B.anthracis)
	Antiphagocytic M protein				Protein involved in beta-1,3-glucan synthesis
					Putative polysaccharide deacetylase
**Quorum sensing / Biofilm formation / Chemotaxis / Siderophores genes**	Methyl-accepting chemotaxis protein I (serine chemoreceptor protein)		Isochorismatase of siderophore biosynthesis	Siderophore biosynthesis non-ribosomal peptide synthetase modules	Protein containing cell adhesion domain
				Pulcherriminic acid synthase	Two component system sensor histidine kinase CiaH
				Uncharacterized MFS-type transporter YvmA	hypothetical YMCA protein
				Transcriptional activator NprR	Response regulator FasA or ComE or BlpR
				Two-component response regulator	RapD
					Metal-binding protein ZinT
**Stress response**	Glutamate decarboxylase			Hypothetical radical SAM family enzyme in heat shock gene cluster, similarity with CPO of BS HemN-type	Transcriptional regulator, Crp/Fnr family

				Cyanate hydratase	glyoxalase family protein, putative
					Low temperature requirement protein A
					Membrane-bound metal-dependent hydrolase YdjM, induced during SOS response
**Anti-fungal genes**	probable endoglucanase			Surfactin synthetase subunit 1	
**carbohydrates and phosphate metabolism genes**		Cellulose synthase catalytic subunit [UDP-forming]		Carboxynorspermidine dehydrogenase	Gluconate permease, Bsu4004 homolog
				Phosphoenolpyruvate-dihydroxyacetone phosphotransferase, ADP-binding subunit DhaL	Gluconate operon transcriptional repressor
				PTS system, mannose-specific IID component	N-acetylmuramoyl-L-alanine amidase, family 4
				Sugar isomerase	Predicted transcriptional regulator of the myo-inositol catabolic operon
				PTS system, mannose-specific IIA component / PTS system, mannose-specific IIB component	2-pyrone-4,6-dicarboxylic acid hydrolase, putative

				Phosphate regulon sensor protein PhoR	
				PhnO protein	
				PTS system, mannose-specific IIC component	
				Putative regulator of the mannose operon, ManO	
				Transcriptional repressor GlcR, DeoR family	
**Utilization of sulfar and nitrogen sources genes**				S-methylmethionine permease	
				Allantoate amidohydrolase	
**Phage associated genes/ Transposable elements**	Phage recombination protein Bet	Phage tail length tape-measure protein T	Phage integrase, site-specific tyrosine recombinase	transposase, IS116/IS110/IS902 family	ankyrin repeat protein
	CRISPR-associated protein, Csd2/Csh2 family	Phage protein ea31	transposase homolog for IS232 [Insertion sequence IS1181]	Phage MazG nucleotide pyrophosphatase/toxin-antitoxin (ACLAME 361)	Transposon Tn7 transposition protein tnsB
	phage-related protein	Terminase small subunit [Bacteriophage A118]		Transposase IS116/IS110/IS902	Tn7-like transposition protein D
	Bacteriophage-related protein	Transposase and inactivated derivatives		Phage minor structural protein	DNA recombinase

	integrase/recombinase, putative			Phage protein (ACLAME 1009)	conjugation protein, TraG/TraD family, (pXO2-16)
	negative regulation of the defective prophage PBSX genes			Phage major tail protein	putative ATPase TraE
	Phage tail fibers			Transposase B from transposon Tn554	phage transcriptional regulator, ArpU family
	CRISPR-associated protein, Csd1 family			Repressor (cro-like) [Bacteriophage A118]	plasmid replication protein, (pXO2-38)
	Phage exonuclease (EC 3.1.11.3)			Phage portal protein	Tn7-like transposition protein A
	CRISPR-associated helicase Cas3			TnpA transposase	transposase for IS1630-like insertion sequence element, putative
	CRISPR-associated protein Cas5			Phage exonuclease (EC 3.1.11.3); Putative phage-encoded enzyme involved in integration-recombination	Probable site-specific serine recombinase-resolvase family protein
	some similarities to phage-related terminase small subunit homolog yqaS			Phage tail length tape-measure protein	Hypothetical SAV0808 homolog, near pathogenicity islands SaPI att-site
	Hypothetical protein, Lmo2306 homolog [Bacteriophage A118]				Replication protein O
	Phage terminase, small subunit				Phage-related integrase/recombinase
	Phage capsid and scaffold				Tn7-like transposition protein C

					prophage LambdaBa02, transcriptional regulator, AbrB family
					Type I restriction-modification system, DNA-methyltransferase subunit M (EC 2.1.1.72)
					Hypothetical SAV0808 homolog, near pathogenicity islands SaPI att-site
**Dormancy and Sporulation**		Spore germination protein GerKB	Small acid-soluble spore protein, alpha/beta family, SASP_4	Spore coat protein S	Small acid-soluble spore protein, alpha-type SASP
				Sporulation-associated protease N-terminal domain protein	
				Spore coat polysaccharide biosynthesis protein spsB	
				COGs COG2843	
				Spore germination protein GerKA	
**Secondary Metabolism**					Predicted 2-keto-4-pentenoate hydratase/2-oxohepta-3-ene-1,7-dioic acid hydratase
					Regulator of polyketide synthase expression
