# Supplementary material for: Comparative genomics of Bacillus cereus sensu lato spp. biocontrol strains in correlation to in-vitro phenotypes and plant pathogen antagonistic capacity
Source: Front Microbiol. 2023 Feb 9;14:996287. doi: 10.3389/fmicb.2023.996287 (PMC9947482; doi:10.3389/fmicb.2023.996287)
Supplement: Supplementary file 3 [file Data_Sheet_3.PDF]

Supplementary Table S2: unique genes detected by RAST among the three plasmids of the Bcsl isolates

Stress response	***Phage associated genes/ Transposable elements***	***Metabolism***	***Antifungal***	***ABC transporter and antimicrobial resistance genes***	***Toxins and Antibiotic synthesis genes***	***Quorum sensing / Biofilm formation / Chemotaxis / Siderophores genes***	***Dormancy and Sporulation***	***Pathogenesis related genes and virulence factors***
Universal stress protein family (S-10)	Tn554-related, transposase C (S-10)	Xanthine enzymes (S-10)	Chitin binding protein (S-25)	SmtA protein (S-10)	Lanthionin biosynthesis (S-10)	Methyl-accepting chemotaxis sensor (S-10)	spore coat polysaccharide synthesis (UW85)	Choline binding protein PcpA (UW85)
ADA regulatory protein (S-25)	Putative transposase InsK for insertion sequence element IS150 (s-10)	CDP-glucose 4,6-dehydratase (S-10)	probable endoglucanase (S-25)	Macrolide glycosyltransferase (S-10)	Vancomycin (S-25)	RNA-binding protein Hfq, Bacillus anthracis type 2 (S-10)	CGEA protein (UW85)	Glycosaminoglycan attachment site (UW85)
Cold shock protein of CSP family (S-25)	Holin (S-10)	Gluconolactonase (S-10)	Phenazine biosynthesis protein PhzF like (S-25)	Undecaprenyl-diphosphatase BcrC , conveys bacitracin resistance (S-10)	cESAT-6-secreted WXG100 domain protein, contains Colicin-DNase domain (UW85)	Siderophore biosynthesis non-ribosomal peptide synthetase modules (UW85)		

	Resolvase (UW85)	Mg/Co/Ni transporter MgtE, (S-10)		ABC-type multidrug/protein/lipid transport system, ATPase component (S-10)	Phage MazG nucleotide pyrophosphatase/toxin-antitoxin (UW85)	N-acyl homoserine lactone hydrolase (UW85)		
	Phage exonuclease (UW85)	Patatin-like phospholipase family (S-10)		ABC-type antimicrobial peptide transport system, ATPase component (S-25)				
	Repressor (cro-like) [Bacteriophage A118] (UW85)	HesA/MoeB/ThiF family protein (S-25)		Barstar-like protein ribonuclease (barnase) inhibitor (S-25)				
		Succinoglycan biosynthesis protein (S-25)		RidA/YER057c/UK114 superfamily, group 1 (S-25)				
		Lactate-responsive regulator LldR in Actinobacteria, GntR family (UW85)		9 different kinds of ABC transporters (UW85)				

				Fosfomycin resistance protein FosB (UW85)				
				penicillin-binding protein (UW85)				
